# Impact of mechanical ventilation on the functional status in patients admitted to the intensive care unit

**DOI:** 10.1186/cc10203

**Published:** 2011-06-22

**Authors:** JA Araújo Neto, RF Bomfim, FB Lima, DA Castro, EB Moura, MO Maia

**Affiliations:** 1 Hospital Santa Luzia, Brasília - DF, Brazil

## Introduction

Many ICU survivors report limitations in physical function that, despite showing slow improvement over time, may be long-lasting. As a complication of critical illness, weakness frequently slows and even dominates the course of recovery from critical illness. Patients requiring mechanical ventilation (MV) often have substantial weakness of the respiratory and limb muscles that further impairs their functional status and health-related quality of life.

## Objective

The aim of this study was to evaluate the impact of the use of MV on the functional status.

## Methods

This is an observational, retrospective and analytical study that included patients aged >18 years who were discharged from ICUs from July 2010 to December 2010. We excluded patients transferred to another hospital and who had not been evaluated by the physiotherapy team at the time of discharge. Functionality was assessed at discharge from the ICU and at discharge from the hospital through the Functional Independence Measure (FIM) scale. The following variables were considered: age, gender, APACHE II, length of ICU, length of stay, length of MV and FIM. We used the normality tests, Mann-Whitney test and Wilcoxon test.

## Results

**Figure 1:**
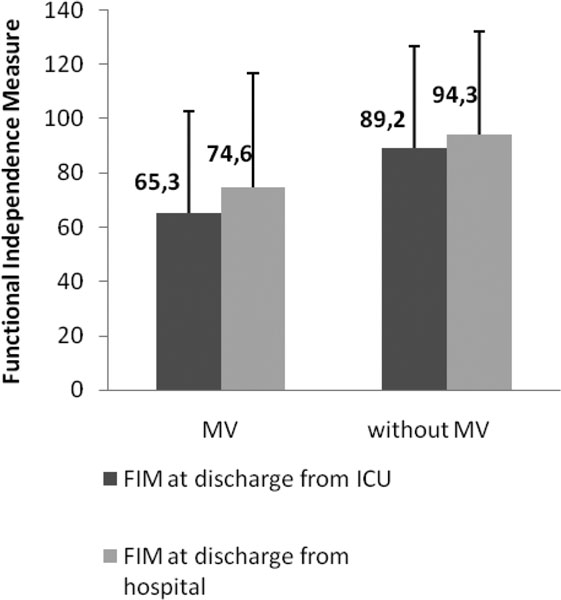
**Values for FIM in patients on MV**. *P < 0.01.

The sample consisted of 158 patients, 51.9% female, mean age 62.5 ± 19.8 years. Of these patients, 30.6% used mechanical ventilation in the ICU. The length of ICU and hospital stay was higher among patients who received MV (length of ICU: 28.3 ± 24.2 days vs. 9.58 ± 16.5 days, *P *= 0.001; length of stay: 37.6 ± 27.4 days vs. 18.9 ± 28.6 days, *P *= 0.001). APACHE II was also higher in this group (13.9 ± 8.3 vs. 10.8 ± 6.57, *P *= 0.02) (Table 1). The functional status was lower in the group undergoing MV at discharge from the ICU (65.3 ± 37.5 vs. 89.2 ± 37.6, *P *= 0.001) and at discharge from hospital (74.6 ± 41.9 vs. 94.3 ± 37.7, *P *= 0.008) (Figure 1).

**Table 1:** Characteristics of the subjects

	MV (*n *= 37)	Without MV (*n *= 121)	*P *value
Age (years)	62.6 ± 17.8	62.4 ± 20.4	0.84
APACHE II	13.9 ± 8.3	10.8 ± 6.57	0.02
SAPS II	38.03 ± 14.4	32.5 ± 11.9	0.02
Length of ICU (days)	28.3 ± 24.2	9.58 ± 16.5	0.001
Length of stay (days)	37.6 ± 27.4	18.9 ± 28.6	0.001
FIM at discharge from ICU	65.3 ± 37.5	89.2 ± 37.6	0.001
FIM at discharge from hospital	74.6 ± 41.9	94.3 ± 37.7	0.008

## Conclusions

In this population we observed that patients submitted to MV have a lower functional status, and higher APACHE II, length of ICU and length of stay.
